# The structure of biodiversity – insights from molecular phylogeography

**DOI:** 10.1186/1742-9994-1-4

**Published:** 2004-10-26

**Authors:** Godfrey M Hewitt

**Affiliations:** 1 Biological Sciences, UEA, Norwich NR4 7TJ, UK

## Abstract

DNA techniques, analytical methods and palaeoclimatic studies are greatly advancing our knowledge of the global distribution of genetic diversity, and how it evolved. Such phylogeographic studies are reviewed from Arctic, Temperate and Tropical regions, seeking commonalities of cause in the resulting genetic patterns. The genetic diversity is differently patterned within and among regions and biomes, and is related to their histories of climatic changes. This has major implications for conservation science.

## Introduction

Phylogeography, named by Avise et al in 1987 [1], is a recent and rapidly developing field that concerns the geographical distribution of genealogical lineages. It grew from the newly acquired technical ability to obtain DNA sequence variation from individuals across a species range, and from this to reconstruct phylogenies. These are then plotted geographically to display their spatial relationships and deduce the evolutionary origins and history of populations, subspecies and species [2,3]. Genetic relationships between species based on polytene chromosome banding patterns had been used earlier to deduce the geographic history of colonization and speciation by Drosophila of the Hawaiian Islands [4], and allozyme variation may still complement DNA information, but the ready access to mitochondrial (mt) DNA sequences opened the door to most animal species and generated this new field [5].

### DNA methods

Whilst mtDNA has lead the way in animal phylogeography, other DNA sequences are used, most commonly chloroplast (cp) in plants and non-coding nuclear (nc) regions in both animals and plants. MtDNA has a relatively fast rate of nucleotide divergence, well suited to examining events over the last few million years, but those of cpDNA and ncDNA are an order of magnitude lower and consequently less useful for such young divergences. More slowly evolving sequences are required for deeper phylogenetic history. For more recent events, like the last 10 thousand years, highly variable markers are needed, such as microsatellites and AFLPs, but whilst useful for population studies they suffer from homoplasy and produce equivocal genealogies [3].

Techniques for obtaining DNA sequence information are still advancing rapidly, with whole genome sequences being produced in a growing number of organisms. This allows sequences and markers to be identified and developed for many types of investigation, and some will be useful for phylogeographic studies. In particular, it is clear that genealogical data is required from several independent nuclear loci to provide a fuller and more reliable history of the species [6]. Single nucleotide polymorphisms (SNPs) are also becoming available across the genome, which will produce comprehensive measures of genetic diversity and allow the construction of better population histories [7].

### Analytical approaches

The advance in DNA technology is producing a wealth of data for individuals, populations and species, and there are concomitant developments in analytical methods to divine demographic history and evolutionary relationships, and to test their significance. This progress in analysis is facilitated by access to increasingly powerful desktop computers on which the increasingly sophisticated software can be used. Haplotype sequences of a particular DNA region can be ordered into a genealogical tree or network, and hence produce their phylogeny. When combined with their population frequency and geographic distribution, this provides a strong basis for inferences on the evolutionary history of the populations and species. The usual phylogeographic approach is to build a phylogeny from haplotype sequences using distance, parsimony and maximum likelihood methods and then represent the lineages geographically. There are several approaches that are regularly used, such as DNA Distance Phylogeography, Nested Clade Analysis, Haplotype Networks, Sequence Mismatch Distribution and Genetic and Demographic Simulation, e.g. PAUP and GeoDis [8-10]. This last approach uses computers to explore broadly how DNA markers evolve in specified molecular, spatial and demographic conditions over history, and is being used increasingly [11]. Recent developments seek to use the genetic data to estimate the demographic history of a population, the dates of historical bottlenecks or expansions, the size of ancestral populations, the location of refugial areas, the dates of divergence, the extent of migration and gene flow, the extent of fragmentation, and the sequence of such events to produce the present geographic distribution of genotypes, e.g. [12-15]. We can expect further developments to provide even more discriminating analyses.

Each DNA sequence has its own genealogy and they may evolve at different rates. Furthermore, the various methods of analysis probe different aspects of the molecular and spatial history. Consequently, to reconstruct a species phylogeographic history one would ideally like to use a range of sequences (including nuclear, cytoplasmic, sex-linked, autosomal, conserved, neutral, high and low mutation rate) and apply a suite of pertinent analyses. This is not easy and often not possible with resources available. However, technological advances for molecules and computers have been explosive in the past decade, making much more detailed analysis possible today than only a few years ago, and this looks set to continue.

### Paleoclimate and Paleobiology

The very different field of paleoclimatology is also experiencing great advances. The results of these are most pertinent to phylogeographic explanations, since they reveal the past environmental conditions and changes that have molded the evolutionary processes producing the present genetic structure. They provide a framework in which the phylogeny may be reconstructed. Past conditions can be deduced from carbon and oxygen isotopes, radiolarian skeletons, pollen grains and other residues from the sea bed, lake bottoms and ice sheets [16]. Novel information sources like insect exoskeletons, coral terraces and stalagmites are adding to this [17-20]. Such records show that earth's climate has been cooling for some 60 my with periodic (21, 41 and 100 kyr) global oscillations producing increasingly severe ice ages through the Quaternary (2.4 my). These involved greatly enlarged ice sheets and surrounding permafrost, and the lower temperatures and reduced water availability caused great changes in the distribution of species as demonstrated by the fossil record [16,21]. Nested within the major 100 kyr cycles are millennial scale oscillations, which can occur rapidly and are often severe [22,23]. Changes of 7–15°C may occur in decades and persist for centuries, as happened most recently in the Younger Dryas (11 kya), where fossils record shifts in the distributions of species.

These major changes in distributions of species occurred latitudinally as the ice sheets advanced and retreated, altitudinally in major mountain regions, and also longitudinally where new dispersal routes became available, as for example the Bering land bridge produced by the lowered sea level. The demographic fluctuations and adaptive challenges produced by such range changes would have had both stochastic and selective effects on the genetic variation and architecture, and the consequences of these can be studied by genetic and phylogeographic approaches. Thus the once distinct fields of paleobiology and phylogeography are now being combined, and have much to tell us about how present biodiversity was structured.

## Fossil and Genetic Signals of Range Changes

With some effort it is now possible to obtain DNA data from specimens across the present range of a species, but fossil data is often more limited or absent. The most useful fossil data are for Europe and North America, which have extensive networks of pollen cores; a few span 3 ice ages (400 kyr), several reach the last interglacial (125 kyr), and a larger number cover back to the last glacial maximum (LGM 23-18 kyr) [21]. There are also some helpful detailed series of beetle exoskeletons [24]; animal bones and plant macrofossils tend to be localized and discontinuous, but are nonetheless useful markers of time and place. Reconstructions of paleovegetation have been made, e.g. [25], which are quite detailed from the LGM to the present, and when coupled with other fossil evidence indicate the extent and rapidity of changes in species distributions.

During the LGM the ice sheets and permafrost extended towards lower latitudes, so that generally species distributions were compressed toward the equator. Boreal species survived south of the ice in North America and Europe, but large areas of the north eastern Palearctic and Beringia remained ice free and some cold-hardy species appear to have survived here. Temperate species survived further south where habitats occurred to which they were each adapted. In Europe the disjunct southern peninsulas of Iberia, Italy and Balkans were particularly important, while in North America many temperate locations occurred around 40°N between the East and West coasts. Nearer the equator the pollen record is not extensive, but conditions were generally drier in the LGM and Tropical habitats were reduced while desert and savanna increased.

As a consequence, the habitats of many Boreal, Temperate and Tropical species were reduced and fragmented and they survived in refugia; but for some their habitats expanded, like those in the tundra and savanna. As the climate warmed after the LGM and the ice retreated, many Boreal and Temperate species were able to expand their ranges, as were some Tropical species. In some cases the refugial populations died out, but particularly in mountainous regions they could survive by ascending with the climate and their niche, as for example in the Alps, Andes, Appalachians and Arusha mountains. Such refugial regions allow the survival of species through several ice age cycles by ascending and descending to track their habitat, e.g. [26].

Such events modify the genetic content and structure of populations within species, and leave some traces for which we may search. Populations, races and subspecies that have been effectively separated for several glacial cycles will show divergence through the accumulation of neutral and possibly selected DNA changes. The extent of this divergence will be proportional to the time of separation. The haplotype tree or network of an evolving DNA sequence will reflect population expansions and contractions. Increasingly these effects can be analyzed, e.g. [27] and placed in some order of occurrence. When the geographic positions of haplotypes are included, a further range of deductions is possible. For example, recently derived populations will contain a sample of the same haplotypes as the parent populations, which combined with paleo-information allows colonization routes to be deduced [28,29]. The extent of distribution of younger haplotypes compared with that of older ones in the tree provides information on the past fragmentation of populations and processes involved in colonization [30]; this can also be combined with paleo-information to deduce the intraspecific phylogeographic history.

## Higher Latitudes – the Arctic

Most phylogeography has concerned Temperate biota [2,26], but recently a number of species from higher latitudes have been analysed in sufficient detail across their range to provide some first genetic insights into their biogeographic history. These include mammals, birds, fish, crustaceans and plants adapted to such cold conditions [31,32]. Table 1 contains some major studies of Holarctic animal species complexes. During the LGM the greatly extended Arctic ice sheets forced such species south, as evidenced by fossil records in Europe and North America. At the same time, large areas of Northeast Asia and the NW corner of North America were covered in permafrost but not glaciated. Fossil evidence suggests that these also contained refugia, particularly Beringia [18,33,34] which with lowered sea level joined Asia and America across the Bering Straits. The different range changes involved would be expected to have various effects on the genetic diversity that may have left marks of their occurrence and extent.

**Table 1:** Animal species with Holarctic ranges showing distinct phylogeographic pattern, with some indication of their possible divergence times, glacial refugia and genetic signals of population history. CA = Circumarctic, HA = High Arctic. PA = Palearctic, NA = Nearctic, BE = Beringia, GL = Greenland, NT = North Temperate.

Species Range	Phylogenetic Divergence (Myr)	Likely Refugia (fossil evidence *)	Genetic signals of range changes	Authors & Reference
*Larus argentatus spp* Herring gull complex CA HA (+EurAsia Lakes)	9 clades <0.5% (0.1–0.3)	Atlantic Aralo-Caspian	Allopatric fragmentation Bimodal mismatch Recent expansions Hybridizations	Liebers et al [96]
*Rangifer tarandus* tundra reindeer CA HA	7 clades 1–2% (0.1–0.3)	Beringia-Asia * W EurAsia* N America	150 kyr expand 15 kyr expand Ragged mismatch	Gravlund et al [97] Flagstad & Roed [98]
*Lemmus *ssp true lemmings HA PA NA	4 clades 3.8–7.9% (0.5–1.0)	Beringia E Asia Siberia* N America*	Expand Few haplotypes Few haplotypes Expand	Fedorov et al [99]
*Dichrostonyx *ssp collared lemmings HA PA NA GL	6 clades 1–7% (0.1–1.0)	Beringia* Arctic Islands E Asia C&W Siberia*	Not to east Expand Low diversity Low diversity	Fedorov & Stenseth [100]
*Microtus oeconomus* root/tundra vole HA PA BE	4 clades 2.0–3.5% (0.2–0.6)	Beringia S Urals* Caucasus C Europe*	Not far Few haplotypes N expansion N expansion	Brunhoff et al [101]
*Microtus agrestis* field vole not HA, PA	3 clades 0.5–5.2% (0.1–0.6)	S Urals* Carpathians* Iberia	Across Asia N&W expansion Not far	Jaarola & Searle [102]
*Lagopus mutus* rock ptarmigan CA HA	7 clades 0.21–1.12% (0.05–0.1)	Multiple, eg Greenland – Beringia/Aleuts Siberia	Several recent Expansions Only 4% diversity within lineages	Holder et al [103, 104]
*Calidris alpina* Dunlin (migrant) CA	5 clades 1.1–3.3% (0.1–0.3)	W Africa Arabia SE Asia C America	W EurAsia Siberia Beringia Canada	Wennerberg [105] Wenink et al [106]
*Daphnia pulex* Waterflea (clonal) CA HA	7 clades 0.5–3% (0.2–1.5)	Periglacial Some more local clones	Mixing, but some N Amer/Eurasian difference	Weider et al [107]
*Troglodytes troglodytes* Winter wren NT not HA EurAsia BE N Amer	6 clades 3.0–8.9% (0.5–1.6)	NW Amer NE Amer NE Asia C Asia, S Europe	Series of glacial vicariances Recent expansions	Drovetski et al [38]
*Cleithrionomys rutilus/glareolus/gapperi* red-backed voles HA PA NA BE	12 clades 1–10% (0.1–1.8)	C Europe* E Asia* Beringia N Amer*	Several colonizations of N Amer across Beringia from Asia	Cook et al [37]

### Distinct Parapatric Clades, Refugia and Range Changes

The phylogeographic structure, in terms of distinct regional DNA clades, is very marked in some species like the lemmings, voles and wren, moderate in the ptarmigan and dunlin, and less in the more mobile waterflea, reindeer and herring gull. The extent of DNA divergence between major clades in small mammals would suggest effective separation of up to 1 Myr, some 5–10 full glacial cycles, with further subdivision for shallower clades in more recent ice ages. In the gull, ptarmigan and reindeer the divergence among clades is low, indicating events occurring in the last or penultimate glacial cycles. Such recent structure would suggest that these species came from or were reduced to a small ancestral population in the late Pleistocene.

**Figure 1:**
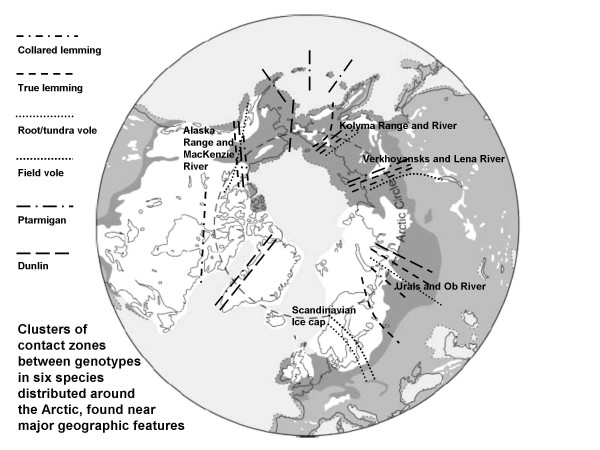
A polar projection showing the general regions of contact between diverged DNA clades of six Holarctic species (see text and references for details and Latin binomials). Note the clustering near features like mountain ranges and major rivers. The Scandinavian cluster, which includes a number of other species, forms where the last remnants of the ice cap melted. Last glacial ice caps and sheets are in white, and tundra is darker grey (I am grateful to Richard Abbott for the basic map).

The deeper clades of the true and collared lemmings, the root and field voles, and to some extent the shallow ones of the ptarmigan and dunlin, are remarkably parapatric and many contacts between them coincide around major features like the Urals, Lena, Kolyma and MacKenzie Rivers (Fig 1) [32]. Regions where several subspecific and sister-specific boundaries coincide, called suture zones [35] have been recorded in North America and Europe, and are probably due to species having similar range changes and refugial areas [29]. Thus these regional parapatric genomes seem to have been diverging separately over a number of ice ages, with distinct refugia from which they colonized to fill their individual interglacial distributions. The pattern of range changes may not be exactly the same through each cycle, but there is no sign of genetic mixing among mtDNA clades, and the major boundary features and refugia are likely to have been similar over the last few ice ages. Other taxon contacts occur in these regions in groups like birds and butterflies, and it will be informative to investigate their phylogeographies to look for commonalties and causes.

Each distinct regional clade would have had its own refugium, but the location of these remains to be determined. There are late glacial fossils of small mammals in Central Europe and also near the southern Urals, which were both parts of the extensive tundra and could have been refugia for European and west Asian clades. An additional refugium in east Asia would account for the clades meeting in the regions of the Lena and Kolyma Rivers, while fossils for a number of species in Beringia indicate that it was probably a significant refugium [32]. A recent detailed examination of the phylogeography of the tundra vole in Beringia [36] pinpoints the contact between the Central Asian and Beringian clades on the Omolon River in the Kolyma uplands, which formed a partially glaciated barrier during the last ice age. This would obviously affect other species and provide the western boundary to many Beringian distributions. The equivalent boundary of Beringia in the east was formed by the North American ice sheets which fringed Alaska from the MacKenzie River to the Aleutian Ranges.

The contractions, expansions and distant colonizations involved in these late Quaternary range changes would have influenced the genetic diversity, and should have left some signs. Low haplotype diversity and shallow clades are expected when populations have been severely contracted, and the age of the subsequent expansion may be gauged by mismatch analysis. The structure of haplotype networks and nested clades also provide indications of such events. Many of the populations and clades reported show these features (Table 1 and references). For example, tundra reindeer, herring gulls and rock ptarmigans have shallow clades diverging in the recent ice ages and populations with mismatch analyses indicating postglacial expansion and colonization of high Arctic regions. In those species like the lemmings and voles where DNA lineage divergence goes back several ice ages to perhaps the onset of more severe glaciations some 0.9 My, most clades are shallow with low diversity, particularly those for Central Asia, suggesting extensive colonization from a much reduced refugial source. This is an emerging feature of some significance. It does seem that even for cold adapted species life was hard in Central Asia during the ice age and it provided few refugia.

### Beringia – an Arctic Refugium

Beringia, which spans Eastern Siberia and Alaska, was united and disjoined each Quaternary ice age by changes in the sea level of some 120 m. It was only partly glaciated and fossil evidence shows it supported a mixture of tundra habitats, which allowed it to act as a causeway for continental migration between Asia and America for various species, including humans. The history of Beringia is currently of interest and some of the lower parts now under the Bering Sea probably had conditions suitable for mesic tundra species even in colder times [18]. Several species have a distinct genetic clade across Beringia and some of these show higher diversity here than where previously glaciated regions were colonized, supporting its role as a glacial refugium [32]. A careful analysis of genetic diversity in the tundra, or root vole, *Microtus oeconomus *through Beringia indicates that the Beringian voles probably expanded from a population with low genetic diversity, which had colonized from Central Asia in the penultimate glacial cycle [36]. A broader phylogeographic study on related taxa of red-backed voles, *Clethrionomys*, demonstrated that North America has been colonized successfully from Asia at least twice, possibly three times in the mid-Pleistocene, and *C. rutilus *reached Alaska quite recently with the first Nearctic fossils in the Holocene [37]. The phylogenies of a number of other species, including *Homo sapiens *also indicate Beringia as a colonization corridor.

Analysis of the DNA differences among these *Clethrionomys *species reveals that they have been diverging from the Early Pleistocene, whilst the divergence within the other listed species is much younger (Table 1). An exception is the winter wren, where there are six clades that appear to have begun diverging some 1.6 Mya, suggesting possible cryptic species [38]. These distinct clades contain low diversity and signs of contraction and expansion. The species does not inhabit truly high Arctic habitats, reaching down to more Temperate latitudes. It would seem that its Holarctic range was progressively broken up by the increasingly severe Pleistocene glaciations, with connection across the oceans, Beringia and the centres of the continents becoming more difficult or impossible as its more Temperate range was forced south.

## Mid Latitudes – Temperate Regions

So called Temperate species span a wide range of latitudes, since the climate determinants like insolation, oceans and altitude may produce suitable habitats between 20° and 60°. In Eurasia and North America those more cold hardy species generally have more northerly distributions than those better adapted to more southerly warmer climes, and this will also be reflected in altitudinal range differences. This would also be true of distributions in glacial periods, so that refugia for north Temperate species were nearer to the ice and permafrost than those of more southerly ones. Fossil evidence is very important in properly determining such Pleistocene range changes [39]. In the Arctic species considered (Table 1), the winter wren and field vole provide examples of low Arctic/north Temperate species in what is a progression of adaptations, niches and ranges on the cold to warm axis from pole to equator.

### Refugia and Colonization in Europe and North America

Paleoclimate and phylogeography have been most researched in Temperate Europe and North America, generating many particular examples and several more general conclusions [2,26]. In particular, the similarity among DNA haplotypes across a species range allows the deduction of which northern populations came from which southern populations, and their likely postglacial colonization routes. The fossil record underpins these deductions, particularly for refugial areas and times of colonization [26,28]. Thus many species survived the LGM in southern Europe, the centre and north being covered in tundra and ice. Fossil and DNA evidence show that the peninsulas of Iberia, Italy, Greece and the Balkans were major refugia and contributed variously to the postglacial recolonization of the north. Whilst a few mobile species crossed from North Africa, the Mediterranean Sea appears to have been a major barrier throughout the Quaternary. For many species these southern refugial areas currently contain much genetic diversity for haplotypes, lineages and subspecific taxa. These southern parts of Europe are also mountainous, so that species may survive by ascending and descending with climate changes. The extent of genetic divergence among these peninsular populations clearly indicates that for many species they have been effectively separate for several to many ice ages. Importantly, this separate refugial survival is seen as a causative factor in divergence and speciation [3,29]. It is possible to deduce pathways of genetic divergence in a geographical framework that has produced the continent's diversity of populations, subspecies and species.

The fossil record, in particular that of pollen and beetles, shows that postglacial colonization was to a large extent a property of the individual species niche and the distribution of its habitat. Nonetheless, the patterns of DNA divergence within species have some common features, which argue for common colonization processes and routes [29]. Temperate species often show reduced haplotype diversity in the north, which is considered to be the result of rapid colonization with repeated founder events. This is seen in many species in Europe and North America, e.g. [28,40-46]. Furthermore the recolonized areas are often a broad patchwork of distinct genomes that have emanated from the different refugia, and which usually form hybrid zones where they make contact. These hybrid zones in different species often appear to be clustered together and so may be considered to belong to suture zones [29,35].

**Figure 2:**
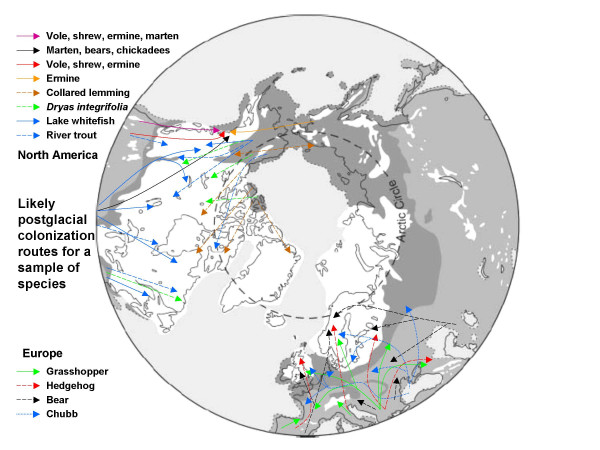
Likely postglacial colonization routes from refugial areas in Europe and North America for a distinctive sample of species that have been deduced from DNA haplotype relationships. Note that regions like central Scandinavia, Britain, the Pacific North West and central Canada contain a mixture of species whose genomes have come from different refugia (see text for discussion).

In Europe the Balkan haplotypes and genomes provided the main source for postglacial colonization for many species, while less came from Iberia and few came from Italy, probably hindered by the ice-capped Pyrenees and Alps. Species that exemplify these different patterns of colonization are the grasshopper, *Chorthippus parallelus*, the bear, *Ursus arctos*, and the hedgehog, *Erinaceus europaeus/concolor *[29]. Freshwater fishes like the chubb, *Leuciscus cephalus*, often show colonization by different haplotypes up the Danube and Dneiper Rivers from the Black Sea (Fig 2) [32]. Many European species phylogeographies are emerging and a considerable number broadly show these distribution patterns and probably followed similar colonization routes despite differences in their niche, mobility and life history. This apparent and remarkable commonality would seem to be a result of colonization following postglacial climate change in Europe's particular geography of southern peninsulas, transverse mountain ranges and northern plains. It demonstrates the explanatory power of combined phylogeography and paleoclimatology.

A particularly interesting consequence of these various colonization routes is that northern regions like Scandinavia and the Pacific NW of America have biotas that are mixtures of species whose genomes came from different refugia [26]. Thus in Central Scandinavia the grasshopper genome came from the Balkans, the bear from Iberia and Russia, the hedgehog from Italy and the chubb from the Black Sea. For the NW corner of America, where Alaska, the Yukon and British Columbia meet, DNA evidence suggests that it has been colonized sequentially from four directions; (1) initially from the south along the coast by the long-tailed vole, dusky shrew, ermine and marten, (2) then from the south by an inland route by all but the marten, (3) from Beringia by the ermine, and (4) from the Appalachians by the marten, and possibly bears and chickadees (Fig 2). Several plant species also colonized probably along the two southern routes [32]. Such mixed biotas carry a number of important implications. They mean that the component species from different refugia have not been evolving together during the previous glacial periods, so any close coadaptation must be either postglacial, or possibly survive from when their distant ancestors were sympatric. More general species-wide coadaptations may be maintained if the different species survive together in refugia. Where genomes from two or more refugia come together, genetic diversity will be increased by the presence of diverged lineages, as seen in hybrid and suture zones. Two populations in the same region living in similar habitats, but from different colonizing refugia will possess very different alleles and genomes, while conversely two very distant populations in distinct habitats may have the same refugial genome. This points to the importance of population history in the process of post glacial adaptation, and our understanding of it.

### Mediterranean Latitudes – 30–40°N

The Mediterranean Sea, with Europe's refugial peninsulas and mountains in the north and North Africa to the south lies between roughly 30°N and 40°N, where at these latitudes in North America there are the Southern States across the Appalachians and Rockies to California. While in Europe the Scandinavian ice sheet came down to Warsaw about 52°N with extensive tundra to the south, the Laurentide ice sheet reached near 40°N. below the Great Lakes, with very little tundra and steppe. Such contrasts in geography have produced differences in the phylogeography of species, but there are also similarities. Species in these southern regions generally contain greater diversity for alleles, populations and subspecies, which in several form distinct geographic genomic patches. The divergence among southern lineages and patches is often deeper than further north, indicating a longer survival and probably in the same region. This southern divergence is often estimated to be over many ice age cycles from the Early Pleistocene or even the Pliocene for some species [26]. The best-studied regions in these latitudes are Iberia [47], South Eastern USA [2] and West Coast USA [48-51].

Molecular phylogeography began in the SE USA [1] and many terrestrial and aquatic species there have marked genetic substructure with concordant genomic boundaries [52]. A number of recent studies in Iberia, covering a range of organisms, e.g. beetles [53], lizards [54], salamanders [55], woodmice [45], rotifers [47], and several plants, also show this intraspecific diversity and substructure, with lineages from as early as the Pliocene and often in mountain regions. Likewise West Coast phylogeography for salamanders [56], woodrats [51], shrews [50], frogs [57] and other species from California and the Cascades [49] reveals geographically structured lineages diverging from the Pliocene and Pleistocene. This pattern and divergence would seem to be a product of the geological and climate changes that have occurred, involving major mountain building and Quaternary ice ages. Lineages and populations from the northern parts of such southern regions, like the southern Appalachians, west and central Iberia and the northern Cascades, appear to have provided the main source for northward postglacial colonization, while genomes to the south survived with altitudinal shifts in broadly the same regions [32].

Species with distributions north and south of the Mediterranean Sea must have managed to cross this now major barrier to terrestrial organisms at some stage before or since the opening of the Gibraltar Straits some 5.3 Mya after the Messinian crisis [58]. If this was before 5.3 My, they are likely to have diverged to sister species if there has been little gene flow. There are now phylogeographic studies on a few species both terrestrial and volant. In terrestrial species of salamanders *Salamandra *spp and scorpions *Buthus *spp [59,60] the DNA data shows that the N African/European divergence is old, before the opening of the Gibraltar Straits. While in the woodmouse *Apodemus sylvaticus *[45] the N African haplotypes appear recently derived from southern Iberia, possibly transferred by humans. Interestingly an old divergence in holm oak, *Quercus ilex*, may also perhaps be due to humans [61]. Five flying species have been examined, of which the chaffinch, bearded vulture and barbastelle bat [62-64] have DNA phylogenies that indicate the Gibraltar Strait has not been a major barrier. In dragonflies *Calopteryx *spp [65] the North African genotypes are related to the Italian ones, and in honey bees [66] some African mtDNA haplotypes are found in south Iberia and Sicily, but nuclear markers indicate little migration. These latter two species and the bearded vulture appear to have crossed the Sicilian Channel, which was also narrow during the lowered sea level of the pleniglacials.

## Lower Latitudes – Tropics and Savannah

Much of Africa, South America, South East Asia and North Australia lie in the Tropics. They are rich in species diversity, but with a few notable exceptions little is known of their phylogeography or their paleobiology [26]. During the recent ice ages the climate was colder and drier in the Tropics, with increased deserts and savannah and reduced rain forests. The pollen record is unfortunately poor, but it seems that forest species descended the mountains (~6°C lower LGM), while lowland forest species may have survived in many local wet places and gullies [16,67,68]. It would seem clear that even tropical biotas have undergone repeated changes as a result of climatic oscillations through the Quaternary [26,69].

### Wet Tropics

Several phylogeographic studies from American and Australian rainforests and a few from Africa and Asia indicate that there is great genetic diversity produced by a complex history often diverging in the Pliocene [26,70]. A nice example of this has been studied in the montane forests of the central Divide of Costa Rica, where a North American salamander *Bolitoglossa *has radiated into tropical Middle America [71]. There are strong allozyme and mtDNA differences between several populations only a few kilometres apart (Dnei 0.18, cytb 4%), and 2 putative species within 10 km (Dnei 0.45, cytb 9%). Such genetic distances indicate divergence from the late Pliocene through the Pleistocene. This has involved several adaptations to elevation zones that would have been amplified by local topographic isolation and climatic oscillations. These amphibians may have peculiar attributes, but phylogeographies of birds and freshwater fish in Middle America are also complex with many lineages [72]. There are few studies yet, but it may be that the phylogeographic status of *Bolitoglossa *is not so unusual. Recently over 100 species of rhacophorine tree frog were described in Sri Lanka using mtDNA in combination with exophenotypic measures, when only 18 were previously known, and despite recent extinctions by Man's activities [73]. This suggests that tropical biotas are not only amazingly diverse but highly structured genetically, both above and below the species level.

Genetic studies in tropical rainforests of SW Amazonia and NE Australia show phylogeographic divergence that is geographically concordant across a number of taxa and also originating in the Pliocene. The first set concern some 35 species of small mammals sampled along the Jurua River, and where for the majority there is a deep phylogenetic divide coincident with the Iquitos Arch. This formed as a bulge in front of the uplifting Andes in the Pliocene creating two basins that filled with sediment. The depth of mtDNA divergence between clades in these two basins places their separation at this time [74]. The Iquitos Arch is also implicated in the phylogeographic structure of the dart-poison frog *Epipedobates femoralis*, which was also sampled along the Jurua River traversing this ancient ridge, and which also has coincident mtDNA divergence (cytb 12%) dating to the Pliocene [75]. Interestingly, the collection sites differed markedly for their haplotypes, particularly in the headwaters region, again suggesting considerable local genetic structure. There has been a multitude of hypotheses proffered to explain the structure of Amazonian diversity, and such molecular phylogeographic approaches are beginning to distinguish amongst them.

The second set of phylogeographic studies involves several birds, reptiles and frogs from the remnant strip of tropical forest in NE Queensland [76]. This wet forest has undergone contraction and fragmentation during the drier colder stages and expanded in the interglacials. These show concordant mtDNA divergence that possibly dates back to the Late Pliocene. This is coincident with the Black Mountain Corridor, a narrow region from which rainforest disappeared in the Pleistocene ice ages producing main north and south refugia. The north and south clusters of haplotypes have various structures, some of which show low diversity probably due to population contractions during the ice age, while others have retained more haplotype diversity possibly by survival in local patches of forest. The rainforest snail *Gnarosophia bellendenkerensis *also shows these main phylogeographic features, and with some finer subdivisions. Its distribution from the LGM to the present has been modeled using current climate envelopes for this snail species mapped onto reconstructed paleoclimate distributions [77]. There is good agreement between the changes in the modeled snail distribution from the LGM to the Holocene and the signals from mtDNA data of refugial locations and expansions. Such an approach provides support for the deductions of both paleoclimatic modeling and phylogeography. Furthermore, the relationship between particular paleoclimatic changes and genetic structure is sharpened by studies on other species from this region that are not adapted to rainforest, like grasshoppers and frogs [78,79]. These species show genetic subdivision that is coincident with other physical features that would provide refugia and barriers commensurate with their lifestyle and climatic history, such as the coastal humidity transition or Burdekin Gap.

The phylogeographic pattern demonstrated by amphibians, reptiles and small mammals, is also found in bird species from tropical Africa and South America, which show old Pliocene lineages in the lowland forest and mixed old and recently diverged clusters in the mountains. This has lead to the proposal that such mountains provided a relatively stable environment through the ice ages and rising mountains, in which older lineages survived and new ones were created [80]. DNA divergence in spinetails from the Andes [81] and greenbuls from East Africa [82] provide evidence of montane speciation through the Pleistocene. Such mountain ranges appear to act as generators and reservoirs of lineages and species, and this probably is a function of their low latitude and topographic variety, which provide warm wet habitats through climatic and altitudinal range changes. The repeated small shifts in distribution driven by climate, along with continued uplifting of these mountains would provide conditions for contraction, selection, expansion and speciation.

### Dry Tropics

There have been a number of recent DNA studies of several larger mammals from Africa that provide some interesting insights into how Pleistocene climatic changes modified their ranges and hence their genetic structure and divergence (Table 2). Whilst sampling such species across Africa is a major task, the threat posed by reductions in their numbers means that considerable efforts are being made to assess their genetic makeup for management and conservation, and many have a useful fossil record. They are not inhabitants of the wet forests, which were reduced during the colder drier glacial periods, but are found largely in the savannah grasslands and woodlands that had a different pattern of contraction and expansion. These habitats increased with the onset of the Pleistocene and its increasing glacial activity (3-2 Mya), with periods of dominance recorded around 1.7 and 1.2 Mya. They show increasing prevalence from 0.6 Mya through the Late Pleistocene, and fossils record the emergence of the associated mammal species and their subspecies since then (see references in Table 2). The DNA data, although not an accurate measure over this time scale, also indicates that these are recent events with most divergences in the last 0.4 My.

**Table 2:** Larger mammal species from across African savannah grasslands and woodlands showing phylogeographic pattern, with some indication of their phylogenetic structure and possible divergence times, refugia in west W, east E, and south S, and genetic signals of colonization and population history. LP = Late Pleistocene, MP = Mid Pleistocene, EP = Early Pleistocene, → = colonization/expansion. MS = mismatch expansion. All studies used d-loop mtDNA; also elephant used cytb and microsats, impala cytb, warthog and dog microsats, and buffalo Y.

Species	Phylogenetic structure	Likely Refugia (fossil evidence *)	Genetic signals of range changes	Reference
Hartebeest *Alcelaphus buselaphus*	3 step clades W→S→E,	W, E subdiv, S, *0.7 My>	S→, E→, some shallow clades	Arctander et al [83]
Topi *Damaliscus lunatus*	3 clades S→E (+ S)	(W), E, S, *0.7 My>	S→, 2 clades, MS E→, shallow clade, MS	Arctander et al [83]
Wildebeest *Connochaetes taurinus*	2 clades, S→E, S structured	E, S, *1.5 My>	S→E, MS in E LP	Arctander et al [83]
Kob (& Puku) *Kobus kobus *sl	3 clades, W→E + S	W, E, S * EP>	W↔E several times, lineage mixing, MP LP	Birungi & Arctander [108]
Greater Kudu *Tragelaphus strepsiceros*	3 clades, E, SW→S→E	E, S, SW isolate *widespread	S→E LP shallow clades diversity S>E	Nersting & Arctander [84]
Impala *Aepyceros melampus*	2 clades, SW, S→E	(E), S SW isolate *widespread	S→E LP network cluster E diversity S>E	Nersting & Arctander [84]
Wild dog *Lycaon pictus*	2 shallow clades, E, S, few haplotypes	(W), E, S	W→E&S?, 340 ky> each clade 70 ky> mobile – mixing	Girman et al [89]
Buffalo *Syncerus caffer*	2 shallow clusters W→E+S S nested in E	W, E, S, * LP	W→E <180 ky E→S twice <130 ky MS LP	Van Hooft et al [85]
Elephant *Loxodonta africana*	3–5 clades W→E+S→W	W, E, S, *EP *LP→	W→S&E, EP W→S&E, MP E→W, LP	Nyakaana et al [109] Eggert et al [88]
Warthog *Phacochoerus africanus*	3 distinct clades W→S→E	W, E, S, *0.78 My> *0.4 My→	3 clades isolated MP by dry climate contract/expand LP	Muwanika et al [87]

**Figure 3:**
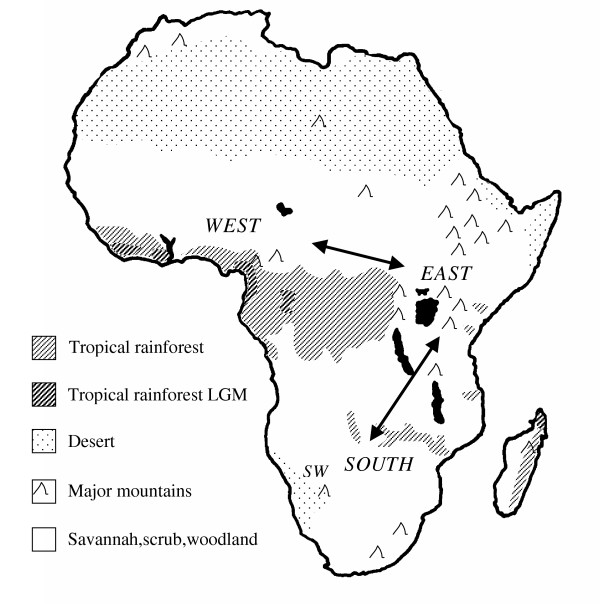
Africa with major vegetation and mountain areas. Reduced Tropical rainforest at the LGM is shown. The Savannah species often show West, East and South clades (see Table 2 for details) and the general areas of these are indicated. The genetic data also indicates colonisations between these possibly refugial areas in the middle and late Quaternary Period.

Most phylogeographies show some 3 major clades that are associated with 3 main areas of Tropical Africa, the west, east and south, indicating that these have been major refugial areas for the development of this divergence through climatic cycles in the Late Pleistocene (Fig 3). Many have shallow clades, mismatch analyses and star-like networks that are the expected result of contractions and expansions of these populations, and their phylogeographies indicate various colonizations between these major regions. In three species, the wildebeest *Connochaetes taurinus*, greater kudu *Tragelaphus strepsiceros *and the impala *Aepyceros melampus*, the data support colonization northwards to the east from refugia in the south of Africa [83,84]. The greater kudu and impala have distinct SW clades, which suggests isolation and survival there, as well as central South Africa, where many species appear to have had a refugium. Interestingly the first wildebeest fossils are from east Africa, so that the species seems to have disappeared from this region and been recolonized recently from the south.

On the other hand, the Cape buffalo *Synercus caffer caffer *has younger haplotypes in the south, which along with mismatch analyses suggest one or two recent colonizations from populations in eastern Africa. The DNA divergence of these from central African buffalo subspecies is perhaps only 180-130 kyr, and roughly coincident with the Cape buffalo's genetic expansion and evidence from fossils [85]. The hartebeest *Alcelaphus buselaphus *and the topi *Damaliscus lunatus *probably survived in a few places in southern and eastern Africa from which they expanded with better conditions [83,86]. The warthog *Phacochoerus africanus *is now relatively widespread, but has 3 distinct clades equivalent to subspecies, and with low divergence within each one [87]. This points to strong isolation through the last few ice ages with considerable recent population reduction followed by population expansion.

The mtDNA phylogeny of the African elephant is more complexly structured, with haplotypes of the putative species of forest *Loxodonta cyclotis *and savannah *L. africanus *elephants mixed together in several clades [88]. It suggests successive production of clades from the early Pleistocene, which involved colonization from the centre to the south and east with increasing savannah habitats, loss of competitors and punctuated by climatic cycles. Such admixture of clades in regions and taxa is probably a reflection of several such colonizations, and a recent invasion of western Africa from central regions is also indicated by the haplotype distribution. The African wild dog is very mobile and populations over the middle part of its current eastern through southern range show a mixture of haplotypes. There are 2 shallow but distinct mtDNA clades that would have diverged perhaps 340 kya, with each coalescing about 70 kya [89]. The cause of this divergence is not clear, but restriction in habitat by climatic oscillations, and separate colonization of east and south from western Africa are possible.

Whilst there are common features within and distinguishing ones between the wet and dry examples reviewed from the Tropics, there are individualities to each species phylogeography; these reflect differences in biology and history that have produced differences in genetic structure. The richness and diversity seen at the species level is multiplied by that within species, and more studies are needed on biotas from such habitats to properly describe and understand them. Little is known from the species rich Tropics of SE Asia, or from the plains of S America and the large areas of Temperate Asia. There are a number of individual studies on a range of species, but one needs several representatives from each community to look for generalities. Given the species diversity in these areas, such genetic and phylogeographic knowledge is particularly important to inform sensible decisions on management and conservation of these resources.

## Lessons for Conservation from Phylogeography

Man is in the unique position to know and predict the consequences for the environment and its biota of his innate will to survive and reproduce. Our actions are greatly modifying both of these, so we face the challenge of managing this sensibly. But we must also think of these natural and induced biotic changes in the light of future major changes in the climate. It is clear that global oscillations, producing great climatic changes, have occurred and will continue; in particular the increasingly severe Quaternary ice ages, which are now well researched and clearly demonstrated. Biotas have changed greatly due to these, and will do so again. We are currently well into an interglacial, the Holocene, and there is debate about how soon it will end and how quick this will be. If the North Atlantic conveyor is turned off, and Man may assist in this, colder conditions may return very quickly. On the other hand, in the shorter term global warming may continue, and Man may assist this! What should we do about biodiversity, and how does phylogeography inform us for this?

The distribution of biodiversity across the world is largely measured as species diversity – their numbers, proportions and distinctness. But within a species there are often several geographic subspecies, and genetic studies have added greatly to knowledge of subspecific diversity, with some regions possessing more lineages and older divergence. Mountain ranges in warm Temperate and Tropical regions are seen to be important because they harbour much diversity at species, lineage and allelic levels. Phylogeographic studies reveal that this is likely a product of species surviving through climatic oscillations by tracking their habitat altitudinally and locally in a varied topography in regions not so affected by the extremes of climate change, e.g. [26]. The southern mountains of Europe, the southern Appalachians and western mountains of USA have clearly been important as refugia and provided most colonists for the vast north Temperate regions today. DNA divergence argues that this has happened repeatedly and so will probably happen again. To date there is little information on the patterns from Asia or S America, and it is needed. Such regions of Temperate refugial genetic diversity have accumulated lineages and alleles through several ice ages and deserve particular research and conservation.

The mountain regions in the wet Tropics of Africa and South America are very rich in species; while phylogeographic studies reveal that these contain divergences often into the Pliocene with subsequent diversification through the Pleistocene. This retention of older diversity through millions of years along with younger lineages argues that they are both generators and reservoirs of divergence and species [26,80]. Less is known about the Tropical regions than Temperate ones, but the phylogeographic evidence does suggest that they can contain greater diversity and divergence in an area, as for example in the wet forests of Costa Rica or Queensland [71,90]. One wonders just what genetic diversity the species in the mountains of China and SE Asia contain. However, tropical Asia is even less studied than Africa and Central America, and with the rapid anthropogenic changes there studies on their phylogeography is urgent. With such information the genetic value of particular regions will be clearer, however their conservation and management involve complex and difficult political matters.

Besides these more general issues, there are a number of more particular lessons and questions. For example, it has recently been noted in several butterflies that the lower genetic diversity produced by postglacial colonization of northern Europe is correlated with their recent decline, as evidenced in national records. Moreover, different deduced postglacial colonization patterns show the same correlation of low genetic diversity and population decline [91]. This suggests that the phylogeography of a species may be used as a predictor of demographic threat and loss. Clearly similar evidence from more species and groups is required to substantiate this.

Another particular example is the genetic diversity created by the formation of a hybrid zone as two genomes meet with postglacial colonization, which is multiplied when several species zones coincide as a suture zone, e.g. [26]. It has been argued that such regions are important because of their genetic diversity [92]. They may well allow the generation of occasional hybrid species [93,94] and possibly reinforcement [95], but except perhaps for climatically very stable locations in the wet Tropics they are transient, and will disappear with each major climatic reversal. They may reform in roughly the same place each cycle, but fossil evidence suggests that this is not necessarily the case [29]. Furthermore, for most zones the diversity they contain is accumulated in their refugia over several cycles, and hence these regions have greater long term value.

The demonstration that the extent of divergence among lineages within and between sister species generally increases from the High Arctic to the wet Tropics reflects their evolutionary age. It can be argued that the richer Tropical biotas are more valuable than the poorer temperate ones, both in terms of their present allelic, lineage and species diversity, and their long term survival. However, this overlooks the particular adaptations of Temperate species and the vast highly productive biotas they produce. The agriculture of the Temperate regions also supports much of the world's population. The consideration of regional diversity and adaptation raises a number of related questions. How well coadapted are recently assembled Temperate ecosystems? Are north Temperate and Arctic species particularly selected for colonization by repeated range change? Are genetically richer genomes more able to adapt to change? Do putative refugial regions contain genetic variation that may be useful in agriculture? And there are many more such considerations.

## Conclusion

The frequent major climatic oscillations in the last 2 My caused repeated changes in the ranges of surviving taxa, with extensive extinction and recolonization in higher latitudes and altitudinal shifts and complex refugia nearer the tropics. As a result of these past dynamics, the genetic diversity within species is highly structured spatially, with a patchwork of genomes divided by often coincident hybrid zones.

The extent of divergence among lineages within and between sister species generally increases from the High Arctic to the wet Tropics and reflects their evolutionary age. Holarctic animal species show shallow but clear phylogeographic structure from the last or recent glaciations. Clades of several species are parapatric near major geographic features like rivers and mountains, suggesting they had similar range changes and refugial areas.

In Temperate regions like Europe and North America there is much more diversity in the south, where it has accumulated in refugia over many ice ages, and much less in the north, where it was lost during postglacial colonization. These northern places have been colonized by species from different southern refugia, and have had little time to become closely coadapted. Furthermore, this loss of diversity in the north is implicated in the present reduction of population abundance in some species. Mammals from the Dry Tropics of Africa often show major clades in the west, east and south indicating major refugial areas for recent divergence through climatic cycles in the Late Pleistocene.

Mountain ranges in warm Temperate and Tropical regions would seem to be important for the survival of lineages through climatic changes, and hence for genome divergence and speciation. Such understanding of the distribution of biodiversity carries serious implications for the theory and practice of conservation.
